# The role of ICT use in reducing social isolation among older adults during the COVID-19 pandemic: evidence from frail and healthy older adults in Sakai City, Japan

**DOI:** 10.1186/s12889-025-23449-x

**Published:** 2025-07-02

**Authors:** Jianyu Huang, Richard Ssempala, Ziyan Wang

**Affiliations:** 1 Graduate School of Economics, Osaka Prefecture University, Naka Ward, Gakuencho, 1-1, Osaka, Sakai, P.O. Box 599-8531, Japan; 2 Department of Economic Theory and Analysis, School of Economics, Makerere University, P.O. Box 7072, Kampala, Uganda; 3 Graduate School of Economics, Osaka Metropolitan University, 3 Chome-3-138 Sugimoto, Sumiyoshi Ward, Osaka, P.O. Box 558-8585, Japan

**Keywords:** Social isolation, Loneliness, Frail older adults, ICT, COVID-19, Aging population

## Abstract

**Background:**

Social isolation among older adults has intensified during the coronavirus disease 2019 (COVID-19) pandemic, disproportionately affecting frail older adults. This study examines the role of information and communication technology (ICT) in reducing social isolation and explores its differential effects between frail and healthy older adults.

**Methods:**

This study employed a quantitative research design with cross-sectional data obtained from the “Survey on Older Adults of Sakai City” in Japan. Social isolation is measured in terms of three dimensions: loneliness, social participation, and access to social support. Ordered probit techniques were employed to estimate the effects of ICT use frequency while controlling for individual characteristics.

**Results:**

Frequent ICT use, including smartphones and voice calls, was significantly associated with reduced loneliness, with frail older adults benefiting more significantly. However, ICT use had a limited impact on increasing the diversity of social participation, likely due to the substitution of in-person activities with online interactions during the pandemic. Access to social support yielded mixed results, with no consistent improvement across the entire sample.

**Conclusions:**

ICT serves as a valuable tool to alleviate loneliness among older adults, especially frail older adults, but it cannot fully replace face-to-face interactions to foster diverse forms of social participation. Simplified ICT tools and tailored training programs are essential to improve accessibility for frail older adults. Future efforts should explore integrating ICT with offline activities and leveraging emerging technologies, such as video conferencing, to address social isolation more comprehensively.

**Trial registration:**

Not applicable.

## Introduction

**Fig. 1:**
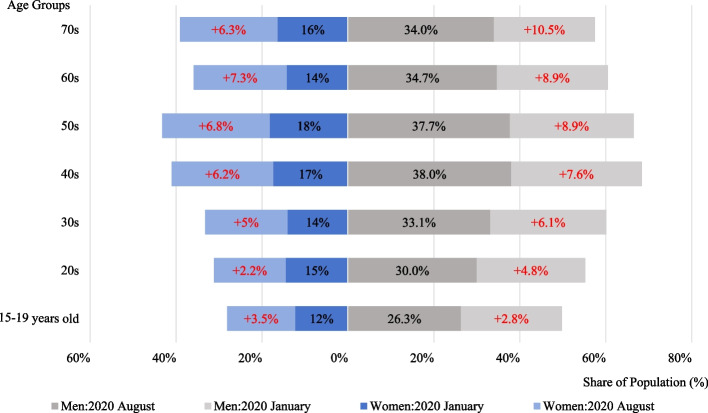
Changes in social isolation by gender and age before and during the COVID-19 pandemic. (Note: This figure compares the proportion of social isolation distributed in January and August 2020 among different age groups. The red text indicates the percentage increase in the population at risk of social isolation in August 2020 compared with that in January 2020 [1]. Source: Tokyo Metropolitan Institute of Gerontology, 2021)

During the COVID-19 pandemic, an online survey on “Differences in Social Isolation by Gender and Age Before and During the COVID-19 Pandemic” was conducted in Japan between August and September 2020 [1]. The survey revealed that the proportion of socially isolated individuals increased across all age groups compared with the prepandemic period. This increase was particularly pronounced among men and older adults (Fig. 1).

In this survey, social isolation was defined as having fewer than one interaction per week with either noncohabiting family members, relatives or friends, or acquaintances. Interactions included face-to-face communication, information exchange (e.g., email or messaging), voice calls, and video calls.

However, whether this standard is comprehensive and reliable remains debatable, as it only measures the frequency of contact and fails to capture the depth and quality of interactions. This study therefore addresses a critical question in regard to whether ICT-based interactions can effectively mitigate social isolation among older adults during global disruptions such as the COVID-19 pandemic. We hypothesize that the frequent use of ICT mitigates loneliness among older adults and has a more pronounced effect on healthy older adults than on their frail counterparts, given the latter’s physical limitations.

### Social isolation risks among older adults under pandemic policies

During the COVID-19 pandemic (hereinafter referred to as the pandemic), Japan implemented strict voluntary self-restraint policies from April 7, 2020, to May 25, 2020, encouraging citizens and businesses to reduce outings and nonessential activities [2]. These policies included measures such as limiting large gatherings, closing entertainment and dining venues, and promoting teleworking, similar to those implemented in many other countries across the globe. In the early stages of the pandemic (January to May 2020), these measures were relatively effective in alleviating pressure on the healthcare system [3, 4]. Although these policies were not legally mandated, social pressure and a sense of collective responsibility motivated public compliance [5]. To prevent the spread of COVID-19, reducing human contact has been deemed essential [6]. This policy has played a crucial role in protecting older adults, who face the highest mortality rates due to COVID-19, from severe health outcomes [6, 7]. However, this raised concerns about losing interpersonal connections and falling into a state of social isolation. Self-restraint policies were implemented multiple times since the outbreak in early 2020 and were gradually relaxed only with the widespread distribution of vaccines. Japan’s voluntary self-restraint policies effectively reduced human contact but simultaneously increased the risk of social isolation, especially among older adults [1].

As self-restraint policies were advanced, specifically those minimizing face-to-face social interactions, many individuals, especially those living alone and older adults, felt isolated and unsupported. For older adults, self-restraint policies bring heightened psychological and physical risks [8]. Studies have shown that social isolation and loneliness significantly increase the prevalence of depression and other psychological issues among older adults [9]. In Japan, where the aging population is a serious societal issue, these policies exacerbate the problems of isolation and solitary living among older adults [10].

Older adults in Japan are particularly vulnerable to the risk of social isolation. On the one hand, the reduction in outdoor activities during the pandemic drastically decreased the social support that older adults typically received through daily activities and social gatherings. On the other hand, self-restraint policies make it more challenging for older adults to access daily life support, such as shopping and medical services. Research indicates that social isolation has severe consequences for the health and quality of life of older adults, even increasing the risk of illness and mortality [11].

### ICT usage, health and social isolation

Social isolation refers to an objective lack of social interaction, whereas loneliness refers to the subjective feeling of being alone [12, 13]. A critical review of the literature reveals a lack of consensus on the definition of social isolation, with some equating it to loneliness and others distinguishing it as a multifaceted construct encompassing reduced social interactions and support [11, 12, 14, 15]. Some scholars equate it directly to loneliness, using the two terms interchangeably [16]. However, most researchers consider social isolation a multidimensional concept related to but distinct from loneliness [14, 17]. For example, studies investigating the impact of ICT on social isolation often focus on its effect on one or more of seven single attributes of social isolation: loneliness, social support, social contact, the number of confidants, social connectedness/social connectivity, social networks, and social well-being. Among these studies, multiple studies have used loneliness as the dependent variable [18, 19].

Notably, researchers conducting qualitative studies have argued that social isolation and loneliness are highly correlated and even interchangeable [20]. For example, Cotten analyzed social isolation and loneliness as two separate outcomes of ICT use and argued that although social isolation and loneliness are not entirely substitutable, they are highly interconnected [21]. Regardless of the definition adopted, social isolation can be seen as the result of reduced social interactions among older adults due to factors such as retirement [18], physical decline (e.g., cognitive and physical disabilities), and shrinking social networks (e.g., divorce or living alone). ICT has the potential to overcome social and spatial barriers to interaction, allowing older adults—often with limited mobility—to engage in simple and cost-effective exchanges and activities through various formats (text, audio, and/or video) anytime and anywhere [22]. Consequently, many researchers have explored its potential to alleviate social isolation among older adults.

In studies investigating the relationship between ICT use and social isolation, most have demonstrated that ICT tools, such as social media, video calling applications, and online communities, have significant intervention effects on social isolation among older adults. For example, Chopik reported that allowing older adults to maintain contact with friends and family via social media helped reduce loneliness and symptoms of depression [23]. Moreover, the positive effects of ICT usage on the mental health of older adults have become increasingly evident [23]. Wang et al. reported in a cross-sectional study that higher frequencies of ICT use were associated with lower levels of social isolation among older adults [15], particularly during the pandemic, when online interaction became a vital way to maintain social connections [24].

In addition, research has shown that ICT interventions significantly increase participants’ social support, social connectedness, and social networks, although they have no effect on the number of close confidants [25]. However, there is no consensus regarding the effectiveness of computers, the internet, and social networking sites in reducing loneliness among older adults. Two randomized controlled trials (RCTs) evaluated the general use of computers and the internet. Slegers et al. targeted healthy older adults living at home [26], whereas White et al. focused on older adults living in subsidized housing or long-term care facilities [25]. Both studies reported no significant effects. Furthermore, another unresolved finding concerns the impact of video conferencing on reducing loneliness among older adults. Blažun et al. reported no change in loneliness levels among participants in long-term care facilities after they used Skype [27]. Conversely, Taiwanese researchers reported a significant reduction in loneliness among long-term care participants using Skype or Windows Live Messenger for video conferencing [28]. Particularly during the COVID-19 lockdown, video communication partially mitigated the emotional burden of loneliness resulting from the lack of face-to-face interaction [29].

Beyond the dimensions of social isolation, some studies have explored the effects of ICT on related constructs, such as physical health and daily activities. ICT use has been found to improve the physical health of older adults [30]. However, its effects on increasing physical activity levels remain inconclusive [26, 31]. According to Chen and Schulz, studies on this topic can be categorized into three types: training-intervention, training-no intervention, no training-no intervention [18]. ICT interventions refer to deliberately designed technological programs (e.g., training courses or device distribution) that aim to improve specific health or social outcomes through externally implemented activities [18, 25]. In contrast, ICT use denotes the spontaneous and voluntary application of technology by individuals in everyday contexts (e.g., routine use of smartphones or social media) without external intervention [21]. Slegers noted that most studies conducted thus far have employed the first two types of intervention experiments. However, these studies often did not report whether efforts were made to ensure that participants did not neglect the intervention tools. Additionally, many of these studies relied on convenience samples with few participants, leading to a high risk of selection bias.

Although there is a substantial body of research focusing on the functional use of ICT (such as internet access, voice communication, and social networking services), studies specifically examining ICT device use (e.g., smartphones, tablets) are relatively limited. Moreover, most existing studies involve interventions or experimental designs, and there is insufficient information on whether assessors or caregivers intentionally intervene during these processes. Consequently, it remains unclear whether any additional or intentional influences were exerted on the study participants by assessors or caregivers [18, 25–27]. Overall, although previous studies on ICT interventions, health, and social isolation have provided valuable evidence of the potential of ICT [12, 15, 18], most of these studies were conducted in controlled experimental settings, limiting the generalizability of their empirical findings. However, during the COVID-19 pandemic, social restrictions forced older adults to rely on ICT tools spontaneously, offering a unique opportunity to observe the effects of technology use in nonintervention contexts [32]. New evidence based on naturalistic surveys and larger sample sizes is needed—an issue this study aims to address [18].

### The focus on frail older adults and group differences

As mentioned earlier, ICT can help alleviate social isolation among older adults to some extent, but its effectiveness is often limited by users’ technical skills and the quality of communication [18]. For example, ICT use does not guarantee communication quality; when ICT-mediated communication is one-sided or ineffective, it may even increase feelings of social isolation among older adults. Therefore, it is crucial to first determine whether older adults are willing and able to benefit from ICT use (e.g., through social media, audio or video calls, and online communities) [21, 33]. Compared with younger individuals, older adults at risk of frailty may face greater challenges in adapting to and operating new technologies, which can affect the extent to which they benefit from communication tools [34]. This issue is particularly significant for frail older adults, who tend to have lower levels of technological competence and are more vulnerable to health risks associated with loneliness [35]. Additionally, owing to physical limitations, frail older adults may rely more on social support than their healthier counterparts do, but they also face greater challenges in adapting to communication tools. This further restricts their opportunities to obtain emotional and social support through these tools [32, 36].

Survey findings during the pandemic have also highlighted gender differences in the degree of social isolation and the effectiveness of technology use [1]. For example, older men are more likely than older women to feel isolated, whereas women tend to exhibit greater adaptability in maintaining social connections. This difference may partially stem from men being less likely to actively seek help or engage in social interactions in daily life [32]. Moreover, while virtual communication can help maintain connections, it may not fully replace the emotional support provided by face‒to-face interactions. Some studies have noted that while virtual interactions such as video calls can alleviate loneliness, their effectiveness in fulfilling deeper emotional needs is limited [37]. For example, research has shown that although communication technologies can help alleviate social isolation, older adults generally prefer face-to-face interactions, a preference that became particularly pronounced during the pandemic [32]. Moreover, owing to the challenges of both technological and psychological adaptation, many older adults reported feeling lonelier during the pandemic. Even when they had access to ICT tools, they often struggled to use and enjoy these tools effectively [35].

### Mechanisms linking ICT use and social isolation

Building on the literature review, we aim to investigate the impact of different types of ICT use on loneliness and social isolation among community-dwelling older adults at risk of frailty, as well as the potential influence of sex on these associations.

One theoretical explanation for this association is that the use of ICT can help older adults maintain and establish social connections, thereby enhancing their social support networks [18, 21, 23, 28, 29]. Within this theoretical framework, different types of ICT use—such as video calls, social media, and online groups—can provide older adults with emotional support, informational support, and companionship, which in turn reduces their feelings of loneliness and risk of social isolation. For older adults at risk of frailty, such social support is particularly important, as physical frailty often limits face-to-face social interactions. ICT tools can partially substitute for and complement traditional in-person communication, thereby alleviating loneliness.

Regarding the potential influence of gender, one explanation is that gender may affect older adults’ acceptance of and preferences for ICT use [20, 24, 32, 34]. For example, older women are typically more focused on social interactions and emotional connections, making them more likely to use communication-oriented ICT tools (e.g., video calls or social media) to maintain connections with family and friends. In contrast, older men may be more inclined to use information-oriented tools, preferring ICT for accessing information and entertainment. These gender differences could influence the effectiveness of ICT use in reducing loneliness and social isolation.

Therefore, it is crucial to examine the role of ICT use in reducing social isolation among older adults during the COVID-19 pandemic, using a large dataset from Japan, a country with a substantial aging population. Research into the role of ICT use is important for guiding practices related to how these developments can contribute positively to people’s health and well-being, especially for older adults in countries with a significant number of such people. Therefore, this study aimed to establish the role of ICT use in loneliness, social participation, and access to social support among older adults, with frail and healthy older adults drawn from Sakai City in Japan.

## Methods

### Data and sample

**Fig. 2:**
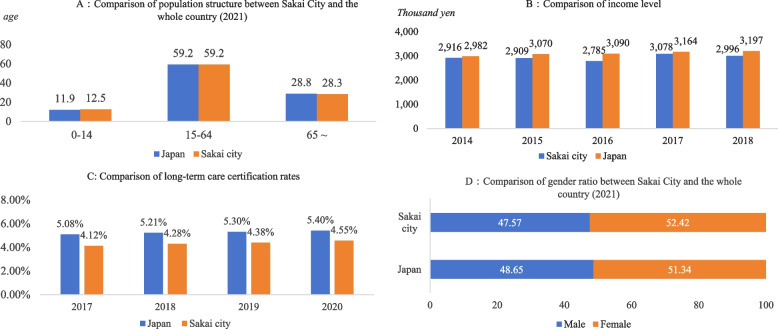
Sakai City’s population structure proportion, education level, gender ratio and income comparison with Japan. (Source: Sakai City: Population by age in all cities and areas (2021) [39]. Population estimation, Statistics Bureau of Japan (2021) [40]. Cabinet Office, National Accounts (GDP Statistics) [41]. Economic Calculation of Sakai Citizen. e-Stat, the Government Statistics Portal of Japan, “Status Report on Long-Term Care Insurance Projects.” [42]; CityPopulation.de, population data provider for Sakai City [43]. **A** Comparison of population structure between Sakai City and the whole country. **B** Comparison of income levels. **C** Comparison of long-term care certification rates. **D** Comparison of the sex ratio between Sakai City and the whole country.)

This study utilized data from the Survey on Older Adults of Sakai City conducted in Osaka Prefecture of Japan in December 2020. The survey provided cross-sectional data on older adult residents in Sakai City, obtained through an official information disclosure request. This dataset is representative, as it reflects the overall characteristics of the Japanese population, including older adults [38]. The demographics of Sakai City in terms of the national averages are shown below (Fig. 2).

The survey aimed to assess the living conditions, health, and welfare of older residents. It included questions covering topics such as the household head’s age and gender, marital status, housing conditions, economic status, family structure, and whether the older adults received care. In this survey, a random sample of 9,400 individuals aged 65 years and above from Sakai City participated in a “fact-finding survey.” The dataset for this study comprises responses from 9,400 individuals and their families who participated in the “Comprehensive Survey on Care Needs for Older Adults in Sakai City” as of December 25, 2020. Due to missing responses, 2,419 samples were excluded, leaving 6,981 valid samples for analysis (*n* = 6,981).

We divided the sample into two groups: frail and healthy older adults. Frailty is currently defined as an independent syndrome in older adults that is characterized by a significant reduction in resistance and adaptive capacity to health problems or external stressors. Fried et al. introduced the “Frailty Phenotype,” defining frailty through five characteristic criteria: unintentional weight loss, fatigue, slowed walking speed, reduced grip strength, and low physical activity levels [44]. An older adult meeting three or more of these criteria is classified as frail. This standard has been widely used in epidemiological and clinical studies to predict health risks in older adults [44].

On the other hand, Rockwood and Mitnitski (2007) proposed the “Frailty Index,” which quantifies frailty on the basis of the accumulation of health deficits, including physical, cognitive, and daily living abilities. This index allows for a comprehensive description of multidimensional vulnerabilities, enabling a detailed assessment of an older adult’s frailty status [45]. Additionally, Gobben et al. introduced a broader definition of frailty, encompassing not only physical but also psychological and social dimensions, such as emotional state and lack of social support [46]. This multidimensional frailty model provides a theoretical foundation for comprehensive evaluations and supports the development of integrated care and intervention plans [46]. In Japan, the Ministry of Health, Labor and Welfare (MHLW) developed assessment scales for identifying frail older adults [47]. Although the Frailty Index and Fried’s Frailty Phenotype are widely used internationally, we adopted the MHLW’s basic checklist, as it is a validated and policy-relevant screening tool in Japan. This checklist enables the classification of frailty on the basis of physical, mental, and instrumental activities of daily living (IADL) indicators, thereby enhancing the applicability of our analysis to the Japanese context and ensuring consistency with national standards.

In this study, we applied the MHLW’s “Basic Checklist” as an assessment tool and evaluated frailty across three dimensions. Frail older adults were defined as those displaying high vulnerability to stressors and health issues, characterized by low IADL, a need for care, and poor mental health. Among the valid samples, the number of healthy older adults was 5,297, accounting for 75.88% of the sample, whereas the number of frail older adults was 1,684, representing 24.12% of the sample.

The proportion of frail older adults aligns with findings from Fried et al., who noted in their study on the frailty phenotype model that approximately 20% of older adults typically met the full criteria for frailty [44]. This consistency reinforces the validity of the 24.12% frailty prevalence observed in our study.

### Measures

Social isolation is widely recognized as a multidimensional construct encompassing both subjective and objective dimensions. Following prior studies [13, 14], we define subjective isolation as the personal perception of being lonely or emotionally disconnected, which we measure via a validated loneliness scale. In contrast, objective isolation refers to observable aspects of social disconnectedness, such as limited participation in social activities or a lack of supportive relationships. On the basis of this framework, this study employs three indicators to capture the multidimensionality of social isolation: (1) loneliness, which represents subjective isolation; (2) the number of types of social participation, which reflects the behavioral aspects of objective isolation (i.e., actual engagement); and (3) the sources of available support, which reflects the structural aspects of objective isolation (i.e., network accessibility).

While previous studies often emphasize a binary distinction between subjective and objective isolation, recent work [14] calls for a refined classification that incorporates both behavioral and structural aspects of social connectedness. In this context, Matthews et al. and Taylor et al. highlighted strong associations and social isolation [19, 48]. The number of types of social participation reflects the breadth and diversity of engagement, serving as an objective measure of social involvement. As shown by Taylor et al. and Xie et al., higher levels of social participation are associated with reduced loneliness and improved psychological well-being. The sources of available support indicators assess the depth of one’s social network by measuring access to emotional and instrumental support [48, 49], with Li et al. [50] emphasizing the critical role such networks play in mitigating social isolation and psychological distress. Building on this theoretical refinement, we conceptualize emotional disconnection, active social engagement, and perceived support availability as interrelated yet distinct dimensions of social isolation. Collectively, these indicators offer a comprehensive assessment that captures the complex and layered nature of social isolation among older adults. Below is a description of the scales for all the outcome variables.

For loneliness, Cotton et al. used a quantitative measurement of loneliness solely as an outcome indicator of social isolation, employing a self-developed scale with three items that asked participants how often they felt distressed for the following reasons: 1. Not having close companions; 2. Not having enough friends; and 3. Not seeing enough close people [21]. This study uses three survey questions as the measurement scale: “How often do you feel you lack companionship?”; “How often do you feel left out?”; and “How often do you feel isolated from others?”. The response options included “rarely”, “sometimes”, and “always”. To avoid overestimating loneliness when all three questions are answered as “2. Sometimes,” we used the maximum value among the three questions to measure the level of loneliness.

To measure the number of types of social participation, we categorized nine social participation activities from the survey into the following four groups: social and community activities, which include participation in volunteer groups, neighborhood associations, and older adult clubs; physical health activities, which include participation in sports-related groups or clubs and health exercises or long-term care prevention salons; hobby and cultural activities, which include hobby-related group activities, participation in study and cultural circles, and activities to share skills and experiences with others; and labor activities, which include income-generating work.

Menec and Adams et al. reviewed the literature on the effects of social and leisure activities on older adults’ well-being and emphasized the importance of regular social participation [51, 52]. Therefore, we categorized the number of types of social participation into four-level variables: “0: None,” “1: Participating in only one type of activity per month,” “2: Participating in two types of activities per month,” and “3: Participating in three or more types of activities per month.”

The measurement of sources of support available reflects the participants’ engagement with different categories of helpers. These categories include spouses, children, siblings, relatives, parents, grandchildren, neighbors, and friends. The variable Sources of Support Available was categorized into four levels: “0: None,” “1: Only one type of person available for support,” “2: Two types of people available for support,” and “3: Three or more types of people available for support.”

The main explanatory variables in this study concern the frequency of ICT use. Four ICT tools were assessed as variables: cell phones, smartphones, tablets, and desktop computers. Moreover, we distinguished two specific ICT functionalities—voice calls and video calls—from general device use and treated them as separate binary variables. This approach enabled us to capture the functional diversity of ICT usage rather than merely reflecting device access or ownership. For example, voice calls can be made via either cell phones or smartphones, whereas video calls are typically conducted through smartphones, tablets, or personal computers. By analyzing these communication functions independently, we aimed to better assess the relationships between specific forms of ICT-mediated interaction and social isolation outcomes. These variables were defined as binary, where “1” indicates use at least once per week, and “0” indicates otherwise [53].

With respect to this definition, Charness and Boot noted in their study on older adults’ technology use that older individuals tend to use devices such as smartphones and tablets less frequently [54]. Therefore, defining “use” on the basis of a specific frequency, such as at least once per week, is considered a reasonable criterion. Additionally, some studies have a dichotomous frequency of ICT use (e.g., “use/nonuse”), which simplifies the model but limits sensitivity to variations in frequency. This can result in the miscalculation of borderline cases, such as by grouping those who use devices biweekly with nonusers, potentially introducing errors into the model. Consequently, the above definition was tailored to the characteristics of this study’s sample [55].

Additionally, the frequency of meeting friends was defined as follows: “At least once a month = 1, otherwise = 0.” Holt-Lunstad et al. noted in a review of social relationships and health that maintaining regular in-person social contact, such as meeting at least once a month, significantly reduces social loneliness and isolation in older adults [56]. Thus, we used this as a key categorical variable to capture changes in social interactions.

In addition to ICT use variables, we controlled for a range of demographic and socioeconomic characteristics commonly associated with social isolation outcomes among older adults [14, 51]. Specifically, we included age (categorized as 65–74 years, 75–84 years, and 85 years or older), gender (female = 1, male = 0), living arrangement (living with others = 1, living alone = 0), and employment status (currently employed = 1, otherwise = 0). Education level was also considered, classified into less than 6 years, 6–12 years, and more than 13 years of schooling. Economic status was grouped into three categories: good, average, and very poor. Additionally, we accounted for health-related behaviors, including drinking status (current drinker = 1, otherwise = 0) and smoking status (current smoker = 1, otherwise = 0). These covariates were incorporated into the regression models to adjust for individual differences that might confound the relationship between ICT use and social isolation indicators. Prior studies have demonstrated that demographic and socioeconomic factors substantially influence both technology adoption and patterns of social engagement among older adults [18, 34, 51].

### Empirical analysis

To examine the relationship between ICT use and social isolation, we employed an ordered probit (OP) regression model. The OP model is appropriate when the dependent variable is an ordinal categorical variable, where the ranking of categories has meaning, but the differences between them are not necessarily equal [57]. In this study, the three key outcome variables—loneliness, number of types of social participation, and sources of support available—are measured as ordered categories, making the OP approach suitable for estimation.

To facilitate interpretation, we report the marginal effects of ICT use on the probability of each category occurring. These marginal effects provide insights into how ICT use influences the likelihood of different levels of loneliness, social participation, and social support.

Additionally, we account for potential heterogeneity between frail and healthy older adults by estimating separate models for each subgroup. The standard errors are clustered at the individual level to adjust for within-sample correlations.

## Results

### Characteristics of the respondents

Table 1 presents the baseline characteristics of the entire sample, as well as subsamples stratified by frailty status. Among the 6,981 respondents, 24.1% were classified as frail, whereas 75.9% were considered healthy. The demographic and socioeconomic covariates clearly differed between the two groups.

**Table 1:** Descriptive statistics for the entire sample and subsamples by health and frailty status

Sample	(i) All		(ii) Healthy		(iii) Frailty	
	Obs.	Percent(%)	Obs.	Percent(%)	Obs.	Percent(%)
**Overall**	6,981	100	5,297	100	1,684	100
**Social Isolation**						
***Loneliness (range 1 ~ 3)***						
1: Rarely	2,824	42.16	2,358	46.12	466	29.38
2: Sometimes	2,992	44.66	2,239	43.79	753	47.48
3: Always	883	13.18	516	10.09	367	23.14
***Number of types of social participation (at least once a month) (range 1 ~ 4)***						
1: None	15	0.24	13	0.28	2	0.13
2: Participates in only one type of activity	288	4.62	247	5.25	41	2.69
3: Participates in two types of activities	1,932	31.01	1,575	33.49	357	23.38
4: Participates in three or more types of activities	3,995	64.13	2,868	60.98	1,127	73.8
***Sources of support available***						
0: None	124	1.89	70	1.39	54	3.47
1: One type	3,252	49.45	2,301	45.84	951	61.12
2: Two types	2,463	37.45	2,023	40.3	440	28.28
3: Three or more types	737	11.21	626	12.47	111	7.13
**Use of Information Communication Tools (ICT)**						
Frequency of meeting friends and acquaintances (at least once a month = 1)	4,282	61.34	3,450	65.13	832	49.41
***Frequency of device use (at least once per week = 1)***						
Cell phone use	1,704	24.41	1,250	23.6	454	26.96
Smartphone use	3,346	47.93	2,814	53.12	532	31.59
Tablet use	692	9.91	599	11.31	93	5.52
PC use	1,381	19.78	1,156	21.82	225	13.36
***Functions and frequency of use (at least once per week = 1)***						
Voice calls	4,810	81.25	3,857	83.92	953	71.98
Video calls	442	8.86	376	9.68	66	5.98
**Individual Characteristics**						
***Age***						
65–74 years	3,372	48.3	2,897	54.69	475	28.21
75–84 years	2,948	42.23	2,091	39.48	857	50.89
85 years and older	651	9.33	302	5.7	349	20.72
***Education***						
Less than 6 years	48	1.16	19	0.62	29	2.74
6–12 years	2,281	55.16	1,560	50.72	721	68.08
More than 13 years	1,806	43.68	1,497	48.67	309	29.18
***Economic status***						
Good (= 1)	880	12.93	745	14.4	135	8.29
Average (= 2)	3,952	58.08	3,081	59.54	871	53.47
Very poor (= 3)	1,972	28.98	1,349	26.07	623	38.24
Employed	1,695	25.72	1,453	28.87	242	15.54
Drinking (= 1)	463	6.73	358	6.86	105	6.32
Smoking (= 1)	741	10.83	564	10.86	177	10.75
Living with others (= 1)	5,562	79.67	4,251	80.25	1,311	77.85
Female (= 1)	3,944	56.51	3,111	58.73	833	49.52

*N* Healthy group = 5297; *N* Frail group = 1684. Frequencies with percentages (%) are displayed in the table

Compared with healthy older adults in the sample, those at risk of frailty were older, had lower household incomes, were less likely to be employed, had lower levels of education, were more likely to live alone, and experienced greater levels of loneliness. Additionally, they reported lower frequencies of using smartphones, tablets, and personal computers.

In terms of age distribution, frail older adults were more likely to belong to the higher age brackets: 50.9% were aged 75–84 years, and 20.7% were aged 85 years or older, compared with 39.5% and 5.7%, respectively, among healthy older adults. In terms of sex, 49.5% of the frail older adults were female, which was slightly lower than the proportion in the healthy group (58.7%).

### Effects of ICT interventions on alleviating social isolation

Before presenting the main results, we first estimate the effects of individual ICT use variables on social isolation by entering each ICT variable separately into the models (details are provided in Appendix A). On the basis of this preliminary analysis, we considered that including multiple ICT variables simultaneously in a single model would be analytically valuable.

However, when multiple ICT use variables are incorporated into a single model, multicollinearity could become a potential concern. To address this, we conducted multicollinearity diagnostics via Pearson correlation matrices and variance inflation factors (VIFs). The results (Appendix B) indicated that multicollinearity was not severe in our joint regression specifications. Furthermore, although certain ICT tools (such as smartphones and tablets) may offer overlapping communication functions, they are not perfect substitutes in the context of older adults. For example, smartphones are more commonly used for real-time interpersonal communication, whereas PCs are utilized primarily for information access or scheduled video calls. Therefore, jointly modeling different ICT tools allows us to better compare their marginal effects and understand their distinctive roles in mitigating social isolation.

To further validate the robustness of the joint regression results, we compared the estimates from the separate regressions presented in Appendix A with those from the joint regressions. We found that the estimated effects of ICT use were generally larger in magnitude in the separate models than in the joint models, which may indicate the presence of omitted variable bias when other ICT variables are excluded. Consequently, to ensure greater model validity and to avoid overstating the effects of any single ICT tool, we chose to present and interpret the main results on the basis of the joint regression models, controlling for all ICT variables simultaneously.

As shown in Table 2, frequent meetings with friends and acquaintances (at least once a month) significantly reduced loneliness, resulting in a strong negative relationship in both frail and healthy older adults. Additionally, the frequency of smartphone use (at least once a week) was significantly negatively associated with loneliness, particularly among frail older adults, where the effect was more pronounced (coefficient = −0.200, *p* < 0.05).

**Table 2:** Relationship between the frequency of ICT use and loneliness

Dependent variable	(i) Healthy		(ii) Frailty	
	Loneliness			
	(1)	(2)	(3)	(4)
**Use of Information Communication Tools (ICT)**				
Frequency of meeting friends and acquaintances (at least once a month = 1)	−0.488***	−0.437***	−0.530***	−0.509***
***Frequency of device use (at least once a day = 1) ***				
Cell phone use	−0.083		−0.293***	
Smartphone use	−0.157***		−0.200**	
Tablet use	0.037		−0.194	
PC use	−0.065		−0.048	
***Functions and frequency of use (at least once a day = 1)***				
Voice calls		−0.355***		−0.284***
Video calls		−0.110		0.208
**Individual Characteristics**				
***Age***				
65–74 years	−0.059	−0.011	0.127	0.115
75–84 years	−0.031	0.017	0.029	−0.008
Education	−0.086*	−0.122**	0.077	0.053
Economic status (Good ~ Poor)	0.185***	0.180***	0.227***	0.233***
Employed	0.030	0.098	−0.079	−0.080
Drinking (= 1)	−0.265*	−0.304*	−0.100	−0.135
Smoking (= 1)	0.101	0.105	−0.016	0.105
Living with others (= 1)	−0.177***	−0.160**	−0.234**	−0.306**
Female (= 1)	−0.246***	−0.234***	−0.156*	−0.174*
Observations	2,780	2,023	935	605

Statistical significance is indicated by *** *p* < 0.01, ** *p* < 0.05, and * *p* < 0.1

In contrast, regarding the usage frequency of other devices, such as tablets and PCs, we found no significant impact on loneliness. The frequency of voice calls also significantly reduced loneliness, with the data indicating that voice calls played a more substantial role in reducing loneliness among healthy older adults than among those at risk of frailty.

Among the covariates, worse economic status was linked to higher levels of loneliness in both groups. Female gender and living with others were associated with lower loneliness, especially among healthy older adults. Higher education was significantly related to less loneliness in the healthy group, but not among the frail. Age, employment, and smoking showed no clear associations, while current drinking had a weak negative effect.

With respect to the number of types of social participation, the results from Table 3 indicate that the frequency of meeting friends had a significant positive effect on the number of social participation types. Moreover, the frequency of smartphone and PC use was negatively correlated with social participation, particularly among frail older adults. The frequency of voice calls also had a significant negative effect on the number of types of social participation, although this effect was weaker among healthy older adults. For video calls, however, we did not observe any significant impact.

**Table 3:** Relationship between the frequency of ICT use and the number of types of social participation

Dependent variable	(i) Healthy		(ii) Frailty	
	Number of types of social participation			
	(5)	(6)	(7)	(8)
**Use of Information Communication Tools (ICT)**				
Frequency of meeting friends and acquaintances (at least once a month = 1)	−0.600***	−0.564***	−0.537***	−0.534***
***Frequency of device use (at least once a day = 1)***				
Cell phone use	−0.013		−0.076	
Smartphone use	−0.667		−0.264**	
Tablet use	−0.027		−0.406**	
PC use	−0.216***		−0.037	
***Functions and frequency of use (at least once a day = 1)***				
Voice calls		−0.159*		−0.409***
Video calls		−0.156		−0.005
**Individual Characteristics**				
***Age***				
65–74 years	0.345***	0.329**	0.444***	0.398**
75–84 years	0.176	0.160	0.107	0.161
Education	−0.048	−0.066	−0.162	−0.254**
Economic status (Good ~ Poor)	0.124***	0.132**	0.073	0.054
Employed	−2.146***	−2.266***	−2.284***	−2.335***
Drinking (= 1)	0.162	0.295	0.303	0.095
Smoking (= 1)	−0.115	−0.105	−0.034	0.143
Living with others (= 1)	0.003	0.023	0.160	0.197
Female (= 1)	−0.214***	−0.191***	−0.110	−0.037
Observations	2,583	1,932	905	592

Statistical significance is indicated by *** *p* < 0.01, ** *p* < 0.05, and * *p* < 0.1

Among the covariates, adults aged 65–74 consistently showed the highest levels of social participation across both groups. Education was positively associated with participation among frail older adults, but not among the healthy. Economic status had a modest positive link with participation in the healthy group. Employment was strongly and negatively associated with social participation in both groups. Female gender was significantly associated with lower participation in the healthy group, while no significant gender difference appeared among the frail (Table 4).

**Table 4:** The relationship between the frequency of ICT use and sources of support available

Dependent variable	(i) Healthy		(ii) Frailty	
	Sources of support available			
	(9)	(10)	(11)	(12)
**Use of Information Communication Tools(ICT)**				
Frequency of meeting friends and acquaintances (at least once a month=1)	0.779***	0.758***	0.816***	0.802***
***Frequency of device use (at least once a day=1)***				
Cell Phone use	0.196***		0.184*	
Smartphone use	0.296***		0.236**	
Tablet use	0.058		0.050	
PC use	0.043		0.057	
***Functions and frequency of use (at least once a day=1)***				
Voice calls		0.513***		0.428***
Video calls		0.074		0.134
**Individual Characteristics **				
***Age***				
65–74 years	0.145	0.057	0.012	0.162
75–84 years	0.117	−0.023	0.183*	0.297**
Education	−0.042	−0.051	0.212**	0.227**
Economic Status(Good ~ Poor)	−0.116***	−0.159***	−0.166**	−0.181**
Employed	0.142***	0.097***	0.031	−0.124
Drinking (=1)	−0.033	0.012	−0.177	−0.377
Smoking (=1)	0.031	0.087	−0.045	−0.011
Living with others (=1)	−0.064	−0.065	0.188*	0.083
Female (=1)	0.703***	0.665***	0.615***	0.548***
Observations	2,762	2,015	914	589

Statistical significance is indicated by ^***^p <0.01, ^**^p <0.05, and ^*^p <0.1

In terms of the number of sources of support, the frequency of meeting friends significantly increased the number of available support sources, indicating that maintaining frequent social connections helps older adults access more sources of support. The frequency of smartphone and cell phone use had a significant positive effect on the number of support sources, particularly among healthy older adults. Additionally, the frequency of voice calls was significantly positively correlated with sources of support, highlighting the importance of maintaining social connections through various forms of communication.

Covariate analysis revealed that economic hardship was strongly associated with having fewer sources of support in both groups. The female gender was consistently linked to greater access to support, while cohabitation showed a marginally positive effect only among frail older adults. In the healthy group, higher educational attainment and current employment were also associated with broader support networks.

As indicated by the marginal effects in Table 5, ICT use is significantly associated with levels of loneliness, social participation, and access to social support among community-dwelling older adults, and these associations vary between the healthy and frail subgroups.

**Table 5:** Marginal effects of ICT use on social isolation

	i) Healthy			ii) Frailty		
	Loneliness(range1~3)	Number of types of social participation	Sources of support available	Loneliness(range1~3)	Number of types of social participation	Sources of support available
	(1)	(5)	(9)	(3)	(7)	(11)
Cell phone use						
1._predict	0.031	−0.000	−0.006***	0.095***	0.000	−0.014*
2._predict	−0.017	−0.001	−0.061***	−0.010**	0.003	−0.046*
3._predict	−0.014	−0.002	0.033***	−0.086***	0.013	0.036*
4._predict		0.003	0.034***		−0.017	0.024*
Smartphone use						
1._predict	0.059***	0.001	−0.009***	0.065**	0.001	−0.018**
2._predict	−0.032***	0.005	−0.093***	−0.007	0.011*	−0.059**
3._predict	−0.027***	0.010	0.049***	−0.058**	0.046**	0.046**
4._predict		−0.016	0.052***		−0.058**	0.031**
Tablet use						
1._predict	−0.014	−0.000	−0.002	0.063	0.002	−0.004
2._predict	0.008	−0.002	−0.018	−0.007	0.017**	−0.012
3._predict	0.006	−0.004	0.010	−0.057	0.070**	0.010
4._predict		0.006	0.010		−0.089**	0.006
PC use						
1._predict	0.024	0.003**	−0.001	0.016	0.000	−0.004
2._predict	−0.013	0.015***	−0.013	−0.002	0.002	−0.014
3._predict	−0.011	0.033***	0.007	−0.014	0.006	0.011
4._predict		−0.051***	0.008		−0.008	0.007
*N*	2780	2583	2762	935	905	914

This table presents the estimated marginal effects from the ordered probit regression models, examining the relationship between ICT use and social isolation indicators among older adults. The columns under “Healthy” and “Frailty” represent the results for nonfrail and frail older adults, respectively. The dependent variables include loneliness, number of types of social participation, and sources of support available. The values under 1._predict, 2._predict, 3._predict, and 4._predict represent the change in the predicted probability of an individual being classified into each category when using ICT tools (e.g., cell phone, smartphone) compared with the reference group (nonusers or lower-frequency users). A positive marginal effect suggests an increased probability of belonging to that category, whereas a negative marginal effect indicates a decreased probability. Statistical significance is indicated by ^***^p < 0.01, ^**^p < 0.05, and ^*^p < 0.1. The sample size (N) varies across models because of missing values

The results indicate that the use of cell phones has a significant effect on reducing loneliness among frail older adults, but not among their healthy counterparts. Specifically, among frail older adults, cell phone use significantly increased the probability of reporting lower levels of loneliness by 9.5% (*p* < 0.001) and decreased the probability of experiencing high levels of loneliness by 8.6% (*p* < 0.001). However, this association was not significant in the healthy group, suggesting that frail older adults, who are more likely to face mobility limitations, may benefit more from cell phone-based communication.

For smartphone use, the results revealed a significant association with lower loneliness levels in both the healthy and frail groups. Among healthy older adults, smartphone use increases the likelihood of reporting low levels of loneliness by 5.9% (*p* < 0.001) and decreases the probability of reporting high levels of loneliness by 3.2% (*p* < 0.001). Similarly, for frail older adults, smartphone use is associated with a 5.8% reduction in the probability of experiencing high levels of loneliness (*p* < 0.01). These findings suggest that smartphones, which offer diverse social functionalities such as video calls and social media, may provide a more effective means of social connection than conventional cell phones do.

The impact of ICT use on social participation varies by health status. While smartphone use does not significantly influence social participation in the healthy group, it has a slight positive effect on increasing participation types among frail older adults. Specifically, frail older adults who use smartphones are 1.7% (*p* < 0.05) to 7.0% (*p* < 0.01) more likely to participate in a greater variety of social activities. This suggests that smartphones may facilitate engagement in social activities even for those with physical limitations.

Interestingly, both cell phone and smartphone use are negatively associated with the number of sources of social support across both health groups. Among healthy older adults, cell phone use decreases the likelihood of having more sources of social support by 0.6% to 6.1% (*p* < 0.001), whereas smartphone use has an even stronger negative effect, reducing social support sources by 0.9% to 9.3% (*p* < 0.001). A similar pattern is observed among frail older adults, where ICT use is linked to a reduction in available social support sources (*p* < 0.05 to *p* < 0.01). These findings suggest a possible substitution effect, where ICT-based communication partially replaces traditional face-to-face interactions and relies on direct social networks.

### Marginal effects by subgroup

Table 6 reports the marginal effects of ICT use functions (voice and video calls) and key demographic characteristics (female, living arrangement, and employment status) on loneliness, the number of types of social participation, and sources of available support across healthy and frail older adult groups.

**Table 6:** Marginal effects by subgroup

	i) Healthy			ii) Frailty		
	Loneliness(range1~3)	Number of types of social participation	Sources of support available	Loneliness(range1~3)	Number of types of social participation	Sources of support available
	(2)	(6)	(10)	(4)	(8)	(12)
Voice calls						
1._predict	0.133***	0.003	−0.016***	0.095***	-	−0.027***
2._predict	−0.078***	0.011*	−0.157***	−0.015**	0.023***	−0.108***
3._predict	−0.055***	0.022*	0.084***	−0.080***	0.068***	0.077***
4._predict		−0.037*	0.089***		−0.092***	0.059***
Video calls						
1._predict	0.041	0.003	−0.002	−0.070	-	−0.008
2._predict	−0.024	0.011	−0.023	0.011	0	−0.034
3._predict	−0.017	0.022	0.012	0.059	0.001	0.024
4._predict		−0.036	0.013		−0.001	0.018
Female						
1._predict	−0.037	0.037***	−0.003	0.027	-	0.008
2._predict	0.021	0.164***	−0.03	−0.004	0.134***	0.031
3._predict	0.015	0.321***	0.016	−0.023	0.388***	−0.022
4._predict		−0.521***	0.017		−0.522***	−0.017
Living with others						
1._predict	0.060**	0	0.002	0.103***	-	−0.005
2._predict	−0.035**	−0.002	0.02	−0.016**	−0.011	−0.021
3._predict	−0.025**	−0.003	−0.011	−0.086**	−0.033	0.015
4._predict		0.005	−0.011		0.044	0.011
Employed						
1._predict	0.087***	0.003**	−0.021***	0.058*	-	−0.035***
2._predict	−0.051***	0.014***	−0.203***	−0.009	0.002	−0.139***
3._predict	−0.036***	0.027***	0.108***	−0.049*	0.006	0.098***
4._predict		−0.044***	0.116***		−0.008	0.075***
*N*	2,023	1,932	2,015	605	592	589

This table reports the average marginal effects from ordered probit models for frail and healthy older adults. The dependent variables are loneliness (range: 1–3), number of types of social participation (range: 1–4), and sources of support available (range: 0–3). Covariates that were not statistically significant were omitted from the table. Marginal effects for ‘Number of types of social participation = 1’ are not reported due to the small number of observations (*n* = 2). Robust standard errors were used for estimation. ***, **, and * indicate significance at the 1%, 5%, and 10% levels, respectively

Voice calls have emerged as a consistently effective tool for mitigating loneliness. Among healthy older adults, those with frequent voice calls were 7.8% less likely to report moderate loneliness and 5.5% less likely to report severe loneliness. The effects were even more pronounced among frail older adults, with the probability of experiencing severe loneliness decreasing by 8.0% among frequent voice call users. Moreover, voice calls significantly increased access to social support, increasing the number of available support sources by 7.7% among frail older adults.

In contrast, video calls largely fell short of expectations. Across both healthy and frail groups, the use of video calls was not significantly associated with loneliness, social participation, or access to social support, suggesting that not all forms of digital communication are equally effective.

Demographic factors also painted a nuanced picture. Being female appeared to confer a distinct advantage in building social connections. Among healthy older adults, women were 2.7% more likely to engage in a greater variety of social activities and 10.8% more likely to report broader support networks. A similar trend was observed among frail older adults, where females had a 9.8% greater probability of accessing diverse support sources.

Living with others also played an important role, particularly for frail older adults. Frail older adults living with others were 8.6% less likely to experience severe loneliness than those living alone. Among healthy older adults, cohabitation increased the likelihood of reporting low levels of loneliness by 6.0%.

Finally, employment status emerged as a double-edged sword. While being employed substantially increased the probability of participating in multiple social activities—by 32.1% among healthy and 38.8% among frail older adults—it simultaneously decreased the likelihood of achieving the highest levels of diverse social participation. This suggests that although employment fosters engagement, it may also constrain the depth or variety of social ties that older adults can maintain.

### Other sources of heterogeneity

To further explore how demographic factors may shape the effects of ICT use, we conducted subgroup analyses on the basis of gender and coresidence status. The key results are summarized in Table 7, with a full listing of the statistical significance of all interaction terms provided in Appendix C.

**Table 7:** Heterogeneous effects of ICT use by gender and living with others

	i) Healthy		ii) Frailty
	Number of types of social participation	Sources of support available	Number of types of social participation
**Gender**			
Frequency of cell phone use(Cell phone use × Female)			0.122(−0.374*)
Frequency of smartphone use(Smartphone use × Female)			−0.609(0.744***)
Frequency of tablet use(Tablet use × Female)			
Frequency of PC use(PC use × Female)	−0.090(−0.334***)		
**Living with others**			
Frequency of tablet use(Tablet use × Living with others)	−0.348*(0.445**)	0.365**(−0.354*)	
Frequency of PC use(PC use × Living with others)			−0.647(0.698*)

The first coefficient in each cell represents the marginal effect of ICT use frequency among the reference group (e.g., male, living alone). The coefficient in parentheses corresponds to the interaction effect between ICT use and subgroup indicators (Female = 1; Living with others = 1). Only statistically significant interaction terms are reported; nonsignificant interactions and covariates are omitted for brevity. *p* < 0.10, **p < 0.05, ***p < 0.01

As seen in the earlier results, the frequent use of smartphones and voice calls consistently alleviated loneliness and, to some extent, increased access to social support. However, when we delved deeper into subgroup differences, more nuanced patterns emerged.

For healthy older adults, PC use appeared to be less beneficial for promoting social participation in women. Specifically, the negative interaction between PC use and female gender (interaction effect = −0.334, *p* < 0.01) suggests that while ICT use generally facilitates engagement, women using PCs are less likely to participate in a wider range of activities than their male counterparts are. Moreover, living with others also influenced ICT outcomes: among healthy individuals, tablet use was associated with reduced social participation (interaction effect = −0.348, *p* < 0.10) but, paradoxically, was linked to a broader network of support sources (interaction effect = 0.445, *p* < 0.05).

Among frail older adults, the dynamics were notably different. Smartphone use, which already showed a strong link with improved outcomes overall, exhibited an even stronger positive effect for women on social participation (interaction effect = 0.744, *p* < 0.01). This suggests that frail women might be particularly adept at leveraging smartphones to stay socially active. Living with others further complicated the picture: tablet use among frail older adults living with other frail older adults increased their social participation (interaction effect = 0.365, *p* < 0.05) but was simultaneously associated with a reduced number of available support sources (interaction effect = −0.354, *p* < 0.10). PC use showed a more straightforward pattern, with living with others linked to more available sources of support (interaction effect = 0.698, *p* < 0.10).

Taken together, compared with the more uniform effects reported in Tables 2, 3, 4, 5, and 6, these findings illustrate that the benefits of ICT use are far from monolithic. Instead, they vary meaningfully by gender, living arrangement, and health status, revealing hidden layers of heterogeneity that would otherwise be obscured in aggregate analyses.

## Discussion

An understanding of the role of ICT use in reducing social isolation is critical, especially in countries with aging populations. ICT tools are an innovative approach that countries have adopted to address biopsychosocial and economic problems effectively [58]. In this section, we discuss the role of ICT use in the three key domains: loneliness, social participation, and social support among older adults in Japan.

### ICT use and loneliness: greater benefits for the frail

The use of ICTs, particularly smartphones and voice calls, was significantly associated with reduced loneliness among older adults, which aligns with the findings of previous studies emphasizing the value of real-time communication for emotional well-being [21, 29]. Notably, the marginal effects analysis revealed that, compared with healthy individuals, frail older adults experienced greater reductions in the probability of loneliness when using cell phones and smartphones. This suggests that ICT may serve as a critical substitute for face-to-face interactions, particularly for individuals facing mobility constraints [18, 32].

Moreover, voice calls demonstrated consistently beneficial effects across both groups, reinforcing the idea that simple and familiar communication methods are particularly effective in alleviating loneliness among older adults. In contrast, tablets and PCs were not significantly associated with loneliness reduction, likely because of usability challenges and lower adoption rates among older populations [34, 54].

Notably, the marginal effects within the subgroups suggest that frail older adults living with others are slightly less likely to report moderate levels of loneliness than their healthy counterparts. This finding may reflect a greater dependence of frail individuals on their core family members for emotional or social support. Furthermore, among healthy older adults, being female is associated with a marginally reduced risk of experiencing higher levels of loneliness, whereas this association is not statistically evident among frail older adults.

### ICT use and social participation: substitution effect and subgroup differences

One of the most notable findings of this study is the negative association between the frequency of ICT use and the degree of social participation. Frequent ICT use was not associated with increased offline engagement; rather, it was correlated with a reduction in the number of types of social activities, particularly among frail older adults. This substitution effect reflects broader societal changes during the COVID-19 pandemic, where digital communication partially replaced traditional face-to-face interactions [15, 24]. Although ICT can facilitate certain forms of engagement, reliance on digital tools may also reduce opportunities for face-to-face interactions, thereby diminishing the quality and diversity of social participation.

The heterogeneity analysis further revealed subtle differences across subgroups. Among healthy older adults, the use of personal computers was associated with a significant reduction in the degree of social participation, particularly among women. This pattern may reflect gender differences in technology adoption preferences, with the substitution effect being more pronounced among older women [34].

In contrast, among frail older adults, smartphone use was associated with a slight increase in the number of social participation types among women, suggesting that mobile technologies may provide frail older females with new channels for social engagement despite physical limitations. However, frail older males may encounter greater difficulties in using ICT to expand their offline activities, potentially exacerbating gender disparities in social participation.

Overall, while ICT can help maintain basic social connections, without complementary programs that actively promote diverse, offline social participation, it may inadvertently contribute to a narrowing of social engagement opportunities. Hybrid approaches that combine virtual and in-person activities are essential for mitigating the substitution effect and sustaining rich, meaningful social lives among older adults.

### ICT use and sources of support available: maintaining, not expanding

Our findings indicate that ICT use had mixed effects on the number of available sources of social support among older adults. While the use of smartphones and voice calls was positively associated with greater access to emotional and instrumental support, other ICT tools, such as PCs and tablets, showed no significant associations. These results align with earlier studies suggesting that simple and direct communication methods are more effective in maintaining social ties among older individuals [15, 21].

However, the expansion of support networks through ICT use was not statistically significant. In fact, frequent ICT users, particularly frail older adults, exhibited a slight decline in the number of available support sources. In contrast to younger people, who frequently expand their networks online [36], older adults tend to use ICT primarily to sustain preexisting social bonds [14].

One possible explanation is that older adults tend to prioritize maintaining strong, emotionally meaningful relationships rather than forming new, weaker ties [24]. This behavioral tendency could limit the ability of ICT to expand an individual’s support network, despite facilitating communication. Moreover, digital communication lacks some of the spontaneity and trust-building dynamics inherent in face-to-face interactions [59]. This further reinforces the view that ICT acts more as a maintenance tool than as a mechanism for social network expansion.

The differential impact across device types also warrants attention. Smartphones and voice calls, which offer immediacy and emotional intimacy, were associated with greater support availability, whereas devices such as PCs and tablets, which often require greater digital literacy and are less communication-centric, did not yield similar benefits. However, heterogeneity analyses revealed an exception: among healthy older adults living with others, tablet use was associated with a greater number of available support sources. This finding suggests that cohabitation with younger family members, such as children or grandchildren, may facilitate older adults’ digital literacy through informal training, thereby enabling them to better leverage ICT tools to expand their support networks. Building on this observation, providing structured ICT training programs for older adults may further maximize the benefits they gain from technology use [36].

### Heterogeneity in ICT effects: the need for tailored strategies

These findings highlight that the impact of ICT use is not uniform, necessitating targeted ICT interventions that consider the specific needs and capabilities of different groups. First, the unequal benefits associated with ICT use underscore the need for tailored digital inclusion strategies. Frequent ICT use was associated with reduced feelings of loneliness, with frail older adults appearing to benefit more substantially. Therefore, tailored interventions such as simplifying user interfaces, implementing ICT training programs, and providing community-based digital literacy support are critical to ensuring equitable access to the emotional and social benefits offered by technology [36].

Second, the observed substitution effect—where increased ICT use was associated with decreased offline social participation—raises concerns. For some older adults, digital communication may replace rather than complement face-to-face interactions [15, 24]. To address this issue, policymakers should promote hybrid models that integrate both online and offline participation opportunities. Examples include community programs offering both in-person and virtual participation options, as well as mobile applications that encourage real-world interactions on the basis of online connections.

Finally, although ICT use may help maintain existing social relationships, it does not appear to expand support networks among older adults, which contrasts with findings observed in younger populations [59]. This highlights the limitations of using digital communication as a substitute for direct social interaction. Strengthening community-based support systems, such as local peer groups or neighborhood initiatives, remains essential for broadening the available support networks for older adults.

## Conclusions

On the basis of these findings, ICT represents a promising tool for addressing social isolation in older adults, but it may not be suitable for all individuals. These findings also contribute to the theoretical understanding of how digital tools can improve older adults'social well-being. ICT use is not just a matter of habit, but a way for older people to stay emotionally connected and socially engaged. It plays a role across different aspects of life—helping reduce feelings of loneliness, supporting participation in daily activities, and maintaining access to social support. This suggests that aging-related policies and frameworks should recognize digital engagement as a valuable resource, especially for those with physical or social limitations. It is therefore necessary for the government to invest in technologies that suit the current and future needs of older adults. Furthermore, implementation research needs to be undertaken with the aim of identifying which subgroups of older adults benefit most from which communication technologies that are available, especially during isolation periods, such as COVID-19, and similar technologies that might be developed in the future. The training of caregivers on the use of these technologies is also paramount for facilitating the engagement of older adults during such periods of need. Additionally, policy interventions should focus on developing voice-activated or one-touch ICT devices to enhance usability for frail older adults, along with community-based ICT training workshops.

## Limitations and areas for further research

In this study, we considered only cell phones, smartphones, tablets and PCs, yet there are other technologies that can be used for social interactions, such as radios and televisions. This might affect the generalizability of our findings, especially in contexts where the use of cell phones, smartphones, tablets and PCs is limited in developing countries [60]. However, in Japan, the use of the considered technologies has a wider range across all age groups, thus providing a stronger basis for our findings [61]. In addition, although we compared the results and conducted robustness checks to address potential concerns, the relatively high correlations among different ICT use variables increase the possibility of residual endogeneity; thus, potential estimation bias cannot be entirely ruled out. Future research could benefit from employing alternative modeling strategies, such as principal component analysis or latent class modeling, to further disentangle the distinct and overlapping contributions of various ICT tools.

Since this study only utilizes data from Sakai City, further research should incorporate data from additional regions to assess potential differences. However, since Sakai City reflects the characteristics of the Japanese population, the present results provide a strong foundation for policy actions.

On the basis of the findings of this study, the next steps should involve utilizing longitudinal data and conducting more carefully designed experimental studies to examine the impact of communication tool interventions on social isolation among older adults. Furthermore, with the rapid advancement of communication technology, the effectiveness of other types of interventions (e.g., mobile applications or YouTube videos) in reducing social isolation should be empirically tested, and the underlying psychological and social mechanisms should be explored [18, 33, 62, 63]. The findings from these studies will contribute to the innovation and effective implementation of communication technology-based interventions for addressing social isolation among older adults.

## Data Availability

The data used in this study were obtained through a formal application and approval process from the survey data of Sakai City, Osaka, Japan. The sharing of research data is restricted and requires permission from the relevant authorities. All the results derived from the data analyses are solely owned by the authors and cannot be publicly shared without authorization.

## Appendix

Appendix A. Robustness checks: separate regressions by ICT device use

We estimated the effect of each ICT device use variable separately by entering them one at a time into the regression models, along with the full set of control variables. Table 8 presents the independent regression results for each ICT device use variable. Separate regressions allow us to preliminarily assess the individual contribution of each ICT device to social isolation outcomes without considering the simultaneous effects of other ICT tools.

**Table 8:** Separate regressions for each ICT device use variable

Variable	i) Healthy			ii) Frailty		
	Loneliness	Number of types of social participation	Sources of support available	Loneliness	Number of types of social participation	Sources of support available
	(1)	(5)	(9)	(3)	(7)	(11)
Cell phone use	0.007	0.062	0.022	-0.212***	0.036	0.092
Smartphone use	-0.168***	-0.152***	0.273***	-0.166**	-0.336***	0.249***
Tablet use	-0.006	0.016	0.113	-0.251	-0.481***	0.126
PC use	-0.093	-0.229***	0.108*	-0.119	-0.178	0.127

This table presents the results from separate regression models where each ICT device use variable (cell phone use, smartphone use, tablet use, and PC use) was entered individually into the models. The regressions were conducted separately for the healthy (i) and frail (ii) older adult groups. Only the coefficients for ICT use variables are reported; coefficients for covariates (including demographic and socioeconomic characteristics) as well as the number of observations are omitted for brevity. All the models controlled for the covariates used in the main analysis. Statistical significance is denoted as follows: ****p* < 0.01, ***p* < 0.05, **p* < 0.1)

We conducted a robustness check by comparing the joint regression results presented in the main text with the results in Table A1. In these separate models, the marginal effects of ICT use on loneliness, social participation, and sources of support were slightly greater. Nevertheless, the robustness check results presented in this appendix support the overall direction of the main findings: ICT use, particularly smartphone use and voice calls, was significantly associated with reduced loneliness and increased social support among older adults.

This pattern suggests the presence of omitted variable bias when analyzing each ICT device separately, as the separate models do not control for the potential overlaps and interactions between different ICT tools. For example, smartphone and tablet use may be correlated but serve different social functions, which can be accurately captured only when jointly modeled. To ensure greater analytical validity and to avoid overstating the effects of any single ICT tool, we chose to base the main analysis on the joint regression models, where all ICT variables were entered simultaneously. By jointly evaluating the effects, we are able to directly compare the marginal contributions of different devices and account for their functional overlaps, particularly among older adults who may use multiple devices for different purposes (e.g., smartphones for real-time communication and PCs for information access).

Appendix B. Multicollinearity diagnostics: correlation matrix and VIF results

Table 9 presents the Pearson correlation coefficients among the main explanatory variables related to ICT use (cell phone use, smartphone use, tablet use, PC use, voice calls, and video calls). The results show that all correlation coefficients are less than 0.5, suggesting that there is no serious multicollinearity concern.

**Table 9:** Pearson correlation matrix among ICT use variables

	Cell phone use	Smartphone use	Tablet use	PC use	Voice calls	Video calls
Cell Phone use	1					
Smartphone use	-0.497	1				
Tablet use	-0.031	0.185	1			
PC use	-0.068	0.284	0.194	1		
Voice calls	0.094	0.240	0.076	0.071	1	
Video calls	-0.069	0.116	0.131	0.048	0.135	1

Variance inflation factors (VIFs) were computed for the main ICT device use variables across three outcome models: loneliness, social participation, and sources of support. All the VIF values are less than 2, and the mean VIF values are approximately 1.37--1.39 across the models, indicating that there are no significant multicollinearity issues. Voice calls and video calls were excluded from the VIF analysis because they served as functional frequency indicators rather than device usage measures. Table 10. Variance inflation factors (VIFs) for the ICT use variables

**Table 10:** Variance inflation factors (VIFs) for the ICT use variables

Variable	VIF		
	Loneliness	Social participation	Sources of support
Cell phone use	1.36	1.36	1.35
Smartphone use	1.72	1.73	1.72
Tablet use	1.1	1.09	1.1
PC use	1.38	1.3	1.3
Mean VIF	1.73	1.80	1.76

This table reports the variance inflation factors (VIFs) for the main ICT device use variables across three outcome models: loneliness, number of types of social participation, and sources of available support. The mean VIF values for each model are also presented

Appendix C. Interaction effects of ICT use with gender and living with others

In this appendix, we present supplementary analyses investigating how the association between ICT use and social isolation outcomes varies by gender and coresidence status. Specifically, we explored whether the effects of different types of device use (cell phones, smartphones, tablets, and PCs) differed between males and females and between individuals living alone and those living with others. The results are summarized separately for frail and healthy older adults across three dimensions of social isolation: loneliness, number of types of social participation, and sources of support available. This appendix aims to complement the main analysis by highlighting potential demographic moderators of ICT effectiveness, providing additional insight into the heterogeneity of digital engagement outcomes during the COVID-19 pandemic (Table 11).

**Table 11:** Interaction effects between ICT use and gender and living with others on social isolation

		i) Healthy			ii) Frailty		
		Loneliness(range1~3)	Number of types of social participation	Sources of support available	Loneliness(range1~3)	Number of types of social participation	Sources of support available
*Gender*							
Cell phone use	×Female (=1)	×	×	×	×	○	×
Smartphone use		×	×	×	×	○	×
Tablet use		×	×	×	×	×	×
PC use		×	○	×	×	×	×
*Living **with others*							
Cell phone use	×Living with others (=1)	×	×	×	×	×	×
Smartphone use		×	×	×	×	×	×
Tablet use		×	○	○	×	×	×
PC use		×	×	×	×	○	×

This table summarizes the statistical significance of the interaction effects between ICT use and gender (Female = 1) or living with others (Living with others = 1) on social isolation, separately for frail and healthy older adults. A circle (○) indicates that the interaction term is statistically significant at the 10% level or better (*p* < 0.10), whereas a cross (×) indicates that the interaction term is not statistically significant

## Abbreviations

ICT: Information and Communication Technology
COVID-19: Coronavirus Disease 2019
MHLW: Ministry of Health, Labor and Welfare
IADL: Instrumental Activities of Daily Living
PC: Personal computer
RCT: Randomized Controlled Trial
